# Supplementary material for: Muscle nonshivering thermogenesis in a feral mammal
Source: Sci Rep. 2019 Apr 23;9:6378. doi: 10.1038/s41598-019-42756-z (PMC6476883; doi:10.1038/s41598-019-42756-z)
Supplement: Supplementary file 1 — Supplementary Info [file 41598_2019_42756_MOESM1_ESM.pdf]

Supplementary Information

Muscle nonshivering thermogenesis in a feral mammal

Julia Nowack[1,3], Sebastian G. Vetter[1], Gabrielle Stalder[1], Johanna Painer[1,2], Maria Kral[2], Steve Smith[2], Minh Hien Le[1], Perica Jurcevic[1], Claudia Bieber[1], Walter Arnold[1], Thomas Ruf[1]

[1]Department of Integrative Biology and Evolution, Research Institute of Wildlife Ecology, University of Veterinary Medicine, Vienna, Austria

[2]Department of Integrative Biology and Evolution, Konrad Lorenz Institute for Ethology, University of Veterinary Medicine, Vienna, Austria

[3] School of Natural Sciences and Psychology, Liverpool John Moores University, Liverpool, UK

Corresponding author: Julia Nowack; contact: J.Nowack@ljmu.ac.uk

Supplemental Experimental Procedure

RNA extraction and reverse transcription

RNA was extracted from 30 mg of muscle tissue using the RNeasy Fibrous Tissue Mini Kit (Qiagen, Hilden, Germany) according to the manufacturer's instructions and included a genomic DNA removal step with *DNaseI*. RNA quantity and quality was determined with Nanodrop 2000c (PEQLAB Biotechnologie GmbH, Erlangen, Germany) with further quality control of the extracted RNA via electrophoresis on a 1% agarose gel (peqGOLD Universal Agarose, PEQLAB). For cDNA synthesis, 1 µg of RNA was reverse-transcribed with MultiScribe™ Reverse Transcriptase (High Capacity cDNA Reverse Transcription Kits, ThermoFisher Scientific) using random hexamer primers.

Primer and probe design

Primer sequences for the target gene, sarcolipin (SLN), were available from Vangheluwe *et al.* (2005) and were checked against the reference mRNA sequence from NCBI (NM_001044566.2). No primer sequence was available for the sarcoplasmic reticulum Ca^{2+}-ATPase (SERCA)1a, so we designed suitable primers from the reference sequence NM_001204393.1 with the assistance of the NCBI primer design tool (Ye *et al.* 2012). Primers for candidate reference genes *Hypoxanthine Phosphoribosyltransferase 1 (HPRT1)* and *Glucuronidase Beta (GUSB)* were available from Charron et al. (2015) and were checked against the NCBI reference sequences (NM_001032376.2 and NM_001123121.1 respectively). Primers were aligned by eye using BioEdit Sequence Alignment Editor [1] and their annealing properties and secondary structures were assessed with NetPrimer (Premier BioSoft). Probe design was performed with the assistance of the PrimerQuest Tool provided by Integrated DNA Technologies (Leuven, Belgium). Probes were located in close proximity to either the forward or reverse primer and designed such that the annealing temperature was a minimum of 5°C higher than either primer and with no guanine base at the 5' end. Target gene probes were labelled at the 5' primed end with FAM and reference gene probes with HEX. Both probe types contained BHQ1 as a quencher at the 3' end.

Droplet Digital™ PCR

Dilution factors for cDNA of 1:10, 1:50, and 1:100 were tested to assess the optimal concentration for Droplet Digital PCR (ddPCR™). A cDNA dilution of 1:50 gave the best balance of positive to negative droplets across test samples. For the reaction setup, 1 µl of the diluted cDNA was used with the Supermix for Probes (No dUTP) according to the manufacturer's protocol (Bio-Rad Laboratories, California, USA). Briefly, the samples were partitioned into droplets before being transferred into a 96-well plate and sealed with sealing foil. Amplification was performed on a GeneAmp® PCR system 9700 (Applied Biosystems, California, USA). Cycling conditions were used according to the manufacturer's instructions (ddPCR™ Supermix for Probes (No dUTP), Bio-Rad). The two reference genes were tested on a subset of samples to determine which was the most stable across treatments. Absolute concentrations of the reference genes were normalized against the input amount of cDNA and the average co-efficient of variation (CV) calculated for each reference across the two treatments (day 1 vs. day 5). The reference HPRT1 displayed the lowest average CV and was used for the gene expression analysis of the two target genes. Data acquisition was accomplished by the QX200™ Droplet Reader (Bio-Rad), and analysed using the Bio-Rad Droplet Digital™ PCR QuantaSoft software. Expression levels are given as the relative ratio of the concentration (copies/µl) of the assay target gene over the concentration of the reference gene.

Table S1: Primer and probe sequences used for gene expression analysis in wild boar piglet samples.

**Primer name**	**Sequence 5'-3'**
SLN_Sus_qPCR_F	GAG AAT GGA GCG ATC CAC CC
SLN_Sus_qPCR_R	ACT TGG CAG CCC TTG AGA GC
SLN_Sus_qPCR_Probe	AGT ATT CAA AGT GCC TCC AGT GCC A
Serca1_Sus_qPCR_F	AAG TGC GGA GAG CAC AAC CC
Serca1_Sus_qPCR_R	ATC GGC AGA GGG TCC ACG TA
Serca1_Sus_qPCR_Probe	CTT TGA GGG TGT GGA CTG TGA GGT C
HPRT1_Sus_qPCR_F	GGC TCC GTT ATG GCG ACC C
HPRT1_Sus_qPCR_R	GAG CAA GCC GTT CAG TCC TGT
HPRT1_Sus_qPCR_Probe	ACC TAA TCA TTA TGC CGA GGA TTT GGA
GUSB_Sus_qPCR_F	GAC GGA CAC CTC CAA GTA CCC
GUSB_Sus_qPCR_R	CGA TGT AGG CGG TAG GCG TG
GUSB_Sus_qPCR_Probe	ACT TCT TCA ACT ACG CGG GAT TGC A

Isolation of muscle SR

Muscle homogenates were prepared from approximately 50 mg muscle tissue. Muscle tissue was washed and minced in ice-cold homogenisation buffer, containing 100 mM Tris (pH 7.0), 250 mM sucrose, 600 mM KCl, 0.5 mM DTT and EDTA-free protease inhibitor cocktail (ROCHE). Tissues were then homogenized in 2 mL of homogenisation buffer by 10-15 strokes with a motor-driven Teflon/glass homogenizer (tube volume, 5 mL, Wheaton, USA) and the homogenizer rinsed with 1 ml of homogenisation buffer (final volume 3 mL).

Homogenates were then centrifuged at 1000 g at 4°C for 20 min to remove cell debris and nuclear fraction. The supernatant was further centrifuged at 15000 g at 4°C for 20 min to remove mitochondria, lysosomes, etc. To the supernatant, KCl was added to a final concentration of 600 mM and centrifuged at 200000 g at 4°C for 30 min. The pellet, containing sarcoplasmatic reticulum microsomes with SERCA protein embedded in the microsomal membranes, was re-suspended in 200 µL of 250 mM sucrose, flash-frozen in liquid nitrogen and stored in LoBind tubes (Sigma/Aldrich) at -80°C. Total Protein concentrations were determined using the Bradford method [2].

SERCA Activity Measurements

SERCA Ca^{2+}-dependent ATPase activities were measured by a standard coupled enzyme assay according to the method previously described by Simonides et al [3]. ATPase activity was coupled to the reaction catalysed by pyruvate kinase which was, in turn, coupled to the reaction catalysed by lactate dehydrogenase. The rate of ATP hydrolysis was calculated from spectrophotometric recording (Hitachi U-1900 Spectrophotometer) of NADH oxidation at 340 nm (ε = 6.25 $mM^{-1}.cm^{-1}$). First, total ATPase activity was measured in the linear region of the progress curve. Then, 100 nM thapsigargin (TG), a specific inhibitor of SERCA [4], was added directly to the assay and the remaining ATPase activity was measured. The difference between ATPase activities recorded before and after TG addition was attributed to SERCA. The standard reaction mixture contained 50 mM imidazole (pH 7.0), 100 mM KCl, 10 mM $MgCl_2$, 10 mM NaN_3, 1 mM EGTA, 0.5 mM DTT, 10 mM phosphoenolpyruvate (PEP), 5 mM ATP, 1 mM $CaCl_2$ (10 µM free Ca^{2+} in solution), 5.3 unit.mL^{-1} pyruvate kinase, 17.5 unit.mL^{-1} lactate dehydrogenase, 300 µM NADH and 2 µM calcium ionophore (A23187) in a final volume of 1 mL. The reaction was initiated by the addition of a volume of the SR sample. ATP and free Ca^{2+} concentrations in the assay were saturating for SERCA, therefore the SERCA ATPase activities were measured at maximum velocity [5]. Assays were performed at a temperature of 37°C. Data presented are means from at least three replicate measurements of SERCA activities, and are expressed as µmol ATP mg^{-1} total protein min^{-1}.

References

1. Hall, T., *A user-friendly biological sequence alignment editor and analysis program for Windows 95/98/NT.* Nucl Acids Symp Ser, 1999. **41**: p. 95-98.
2. Bradford, M.M., *A rapid and sensitive method for the quantitation of microgram quantities of protein utilizing the principle of protein-dye binding.* Analytical Biochemistry, 1976. **72**(1): p. 248-254.
3. Simonides, W.S. and C. van Hardeveld, *An assay for sarcoplasmic reticulum Ca^{2+}-ATPase activity in muscle homogenates.* Analytical Biochemistry, 1990. **191**(2): p. 321-331.
4. Lytton, J., M. Westlin, and M.R. Hanley, *Thapsigargin inhibits the sarcoplasmic or endoplasmic reticulum Ca-ATPase family of calcium pumps.* Journal of Biological Chemistry, 1991. **266**(26): p. 17067-17071.
5. Dode, L., et al., *Low temperature molecular adaptation of the skeletal muscle sarco(endo)plasmic reticulum Ca^{2+}-ATPase 1 (SERCA 1) in the wood frog (Rana sylvatica).* Journal of Biological Chemistry, 2001. **276**(6): p. 3911-3919.

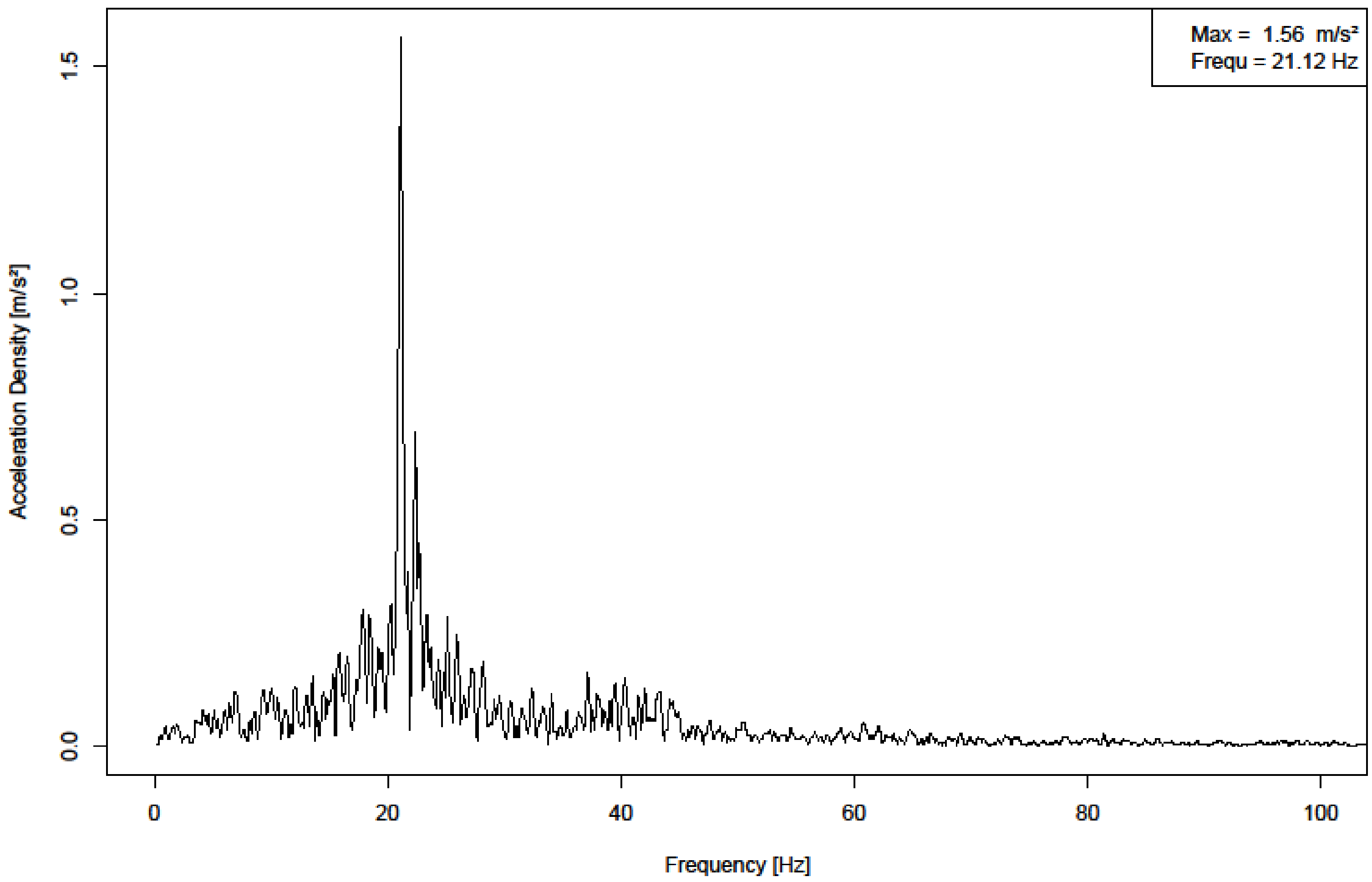

Figure S1: Power spectrum generated from accelerometer data.
